# Is Post-Reprocessing Microbiological Surveillance of Duodenoscopes Effective in Reducing the Potential Risk in Transmitting Pathogens?

**DOI:** 10.3390/ijerph17010140

**Published:** 2019-12-24

**Authors:** Maria Luisa Cristina, Marina Sartini, Elisa Schinca, Gianluca Ottria, Chiara Dupont, Palmira Bova, Gianni Coccia, Beatrice Casini, Anna Maria Spagnolo

**Affiliations:** 1 Department of Health Sciences, University of Genova, Via Pastore 1, 16132 Genova, Italy; maria.luisa.cristina@galliera.it (M.L.C.); elisa.schinca@galliera.it (E.S.); gianluca.ottria@galliera.it (G.O.); lioa@unige.it (C.D.); anna.maria.spagnolo@galliera.it (A.M.S.); 2 S.S.D. U.O. Hospital Hygiene, E.O. Ospedali Galliera, 16128 Genova, Italy; 3 S.C. Gastroenterology, E.O. Ospedali Galliera, 16128 Genova, Italy; palmira.bova@galliera.it (P.B.); gianni.coccia@galliera.it (G.C.); 4 Department of Translational Research and New Technologies in Medicine and Surgery, University of Pisa, 56126 Pisa, Italy; beatrice.casini@med.unipi.it

**Keywords:** duodenoscopes, infection control, post-reprocessing, microbiological surveillance

## Abstract

*Background:* The use of a contaminated endoscope may lead to infections due to the transmission of potential pathogens from patient to patient. *Methods:* Post-reprocessing microbiological surveillance of four duodenoscopes was carried out over a three-year period in the Digestive Endoscopy Unit of an Italian hospital. Sampling of duodenoscopes was performed after the devices have been reprocessed. The initial phase of surveillance involved the contemporary evaluation of the four duodenoscopes; afterwards, microbiological surveillance proceeded at monthly intervals. *Results:* The initial phase of surveillance revealed that three duodenoscopes presented a high level of contamination with “high-concern” micro-organisms, some of which were multi-drug-resistant. The highest values of contamination regarded the species *P. aeruginosa* (2500 CFU/duodenoscope), *K. pneumoniae* (2580 CFU/duodenoscope), and *A. baumannii* (2600 CFU/duodenoscope). Since the cultures were repeatedly positive on three successive occasions, the contaminated devices were sent to the manufacturer for evaluation. Audits were carried out with the personnel responsible for reprocessing, which was aimed to optimize the procedures used, and subsequently, only one case of non-conformity was found. *Conclusions:* Our study highlighted both the potential risk of transmitting pathogens through the use of duodenoscopes and the importance of implementing a well-structured system of microbiological surveillance and training programs, in order to reduce the risk of spreading retrograde cholangiopancreatography (ERCP)-associated infections.

## 1. Introduction

The use of endoscopic procedures, for both diagnostic and therapeutic purposes, is now consolidated and increasing in several specialties of modern medicine [1]. Such procedures involve the use of reusable devices (duodenoscopes, colonoscopes, etc.), which must be properly reprocessed in order to eliminate contamination through biological fluids. During an endoscopic investigation, both internal and external surfaces of endoscopes are exposed to body fluids and potential contaminants [2].

The use of a contaminated endoscope may lead to infections due to the transmission of potential pathogens from patient to patient [3,4]. Outbreaks of healthcare-associated infections are more frequently caused by contaminated endoscopes than by any other medical devices [5,6]. Endoscopy-related infections may occur if micro-organisms are spread through contaminated equipment, or during endoscopy from the gut lumen through the bloodstream to susceptible organs, adjacent tissues, or prostheses [7].

Bacterial and viral outbreaks causing disease and death following gastrointestinal endoscopy have been reported (albeit infrequently) during the past 30 years, particularly after retrograde cholangiopancreatography (ERCP). Epstein et al. reported a cluster of New Delhi metallo-β-lactamase (NDM)–producing *Escherichia coli* infections associated with ERCP [8,9]. Virtually every one of these outbreaks was attributed to one or more breaches of infection control [10,11], particularly due to improper endoscope reprocessing, including failure to clean the instrument channel of the gastrointestinal endoscope with a brush.

Manual cleaning is critical, due to the complex structure of these devices, which have narrow lumina and multiple internal channels. When an endoscope is reprocessed, manual cleaning is followed by high-level disinfection (HLD) treatments, rinsing, and drying, before the device is placed in a dedicated storage locker. Any residual organic material that has not been completely removed during manual cleaning can reduce the efficacy of HLD [5]. Inadequate cleansing, combined with the ability of bacteria to form biofilms on the internal surfaces of the channels, can cause the decontamination process to fail, with the result that the risk of cross-transmission of ERCP-related infections cannot be ruled out. Adequate post-reprocessing microbiological surveillance is therefore strongly recommended by various international guidelines, including those of the Center for Disease Control and Prevention (CDC) [9,12,13,14].

In the present study, post-reprocessing microbiological surveillance of duodenoscopes was carried out over a three-year period in the Digestive Endoscopy Unit of an Italian hospital.

## 2. Materials and Methods

### 2.1. Setting

The Digestive Endoscopy Unit, in which the study was conducted, performs about 350 endoscopic retrograde cholangiopancreatography (ERCP) procedures per year: 2 per week, about 30 per month, by means of 4 duodenoscopes (Olympus TJF145, Olympus Europa SE & Co. KG, 20097 Hamburg, Germany).

Reprocessing of the gastrointestinal endoscopes is carried out in accordance with the indications contained in the international guidelines [7,15,16,17,18] and involves the following phases: Pre-washing; leak testing; manual cleaning; rinsing after manual cleaning; visual inspection; high-level disinfection (automatic by means of peracetic acid); rinsing after high-level disinfection; forced-air drying and storage.

### 2.2. Study Procedure

From April 2017 to October 2019, 124 microbiological samples were taken from the four duodenoscopes (62 from the distal end and 62 from the instrument channel) following post-reprocessing.

The CDC protocol states that, in the event of microbiological non-conformity of three or more repeated samples (due to the presence of any micro-organism of high concern or a microbial load ≥10 CFU/duodenoscope of low-concern micro-organisms), the device must be returned to the manufacturer for the necessary testing. Thus, in order to avoid interrupting the activity of the Endoscopy Unit, we sampled each duodenoscope three times during the same day, each time after a reprocessing procedure.

In the initial phase of the study (Time T0), microbiological surveillance was carried out on all 4 duodenoscopes (a total of 24 samples) in order to ascertain the efficacy of reprocessing. Three of the duodenoscopes repeatedly failed microbiological testing and, as recommended by the CDC protocol, were returned to the manufacturer. In addition, the staff responsible for reprocessing underwent an auditing procedure to detect and correct the failures in the various phases of reprocessing.

The duodenoscopes that had been sent back to the manufacturer were returned to the hospital, where they were again reprocessed. Once they had passed microbiological testing, they were again used for ERCP.

In the second phase of the study (Time T1), microbiological surveillance proceeded at monthly intervals; the duodenoscopes were analysed in turn, one at a time, with 66 samples being taken (33 from the distal end and 33 from the instrument channel).

The third phase (Time T2) was carried out in January 2019, following the substitution of some equipment/apparatuses (e.g., endoscope washers and storage lockers). Sampling was again performed at monthly intervals, with a further 34 samples being taken: 17 from the distal end of the device and 17 from the instrument channel.

### 2.3. Modality of Sampling and Microbiological Analysis

Sampling was carried out in sterile conditions, after the duodenoscope had been reprocessed (after drying); samples were taken from the instrument channel and the distal end of the duodenoscope (elevator mechanism, elevator recess, and elevator channel for duodenoscopes with sealed elevator wire channels).

The cultures from duodenoscopes were examined for two types of microbial growth: High- and low-concern organisms.

High-concern organisms are those most often associated with disease, such as Gram-negative bacteria (e.g., *Escherichia coli, Klebsiella pneumoniae* or other Enterobacteriaceae, and *Pseudomonas aeruginosa*), *Staphylococcus aureus*, and *Enterococcus spp.* Low-concern organisms are less often associated with disease and may arise from contamination of cultures during collection (e.g., coagulase-negative staphylococci, excluding *Staphylococcus lugdunensis*, *Bacillus* species, diphtheroids). [9]

For surveillance, sampling, and microbiological analyses, the 2015 CDC protocol was implemented. Between the two methods of analysis suggested by the CDC protocol, the quantitative method (Membrane Filtration) was used for microbiological analyses. [9,12,13].

In addition, antibiotic resistance was evaluated by means of VITEK^®^ (Biomerieux, Marcy-l’Étoile, Francia).

Microbiological analysis of the water from the last rinse by the endoscope washer was carried out in accordance with the method and modalities indicated by the National Health Service, England (NHS) [19].

## 3. Results

The initial phase of surveillance carried out on the four duodenoscopes revealed 75% non-conformity with regard to high-concern micro-organisms, both in the samples taken from the distal end and in those taken from the instrument channel.

Specifically, samples from the distal end displayed contamination by *P. aeruginosa*, *K. pneumoniae*, *K. oxytoca*, *S. maltophilia*, *A. baumannii*, *E. coli*, and *C. freundii*, while those taken from the instrument channel showed the presence of *P. aeruginosa*, *K. pneumoniae*, *Enterobacter spp.*, *S. maltophilia*, and *A. baumannii*. The highest values of contamination regarded the species *P. aeruginosa* in samples from both sites (2500 CFU/duodenoscope), *K. pneumoniae* in those from the instrument channel (2580 CFU/duodenoscope), and *A. baumannii* at both sampling sites (2600 CFU/duodenoscope) (Table 1).

With regard to the distal end, the antibiogram revealed that 60% of the samples positive for *P. aeruginosa* contained strains resistant to multiple antibiotics (including carbapenems); moreover, *K. pneumoniae* ESBL+ was detected in 40% of the samples positive for this micro-organism.

Regarding the instrument channel, the percentages of samples positive for multi-resistant *P. aeruginosa* and for *K. pneumoniae* ESBL+ proved to be 40%, and 16.67%, respectively. Moreover, 1 sample from the instrument channel was positive for a low-concern micro-organism (*P. luteola*), the concentration of which, however, was 450 CFU/duodenoscope.

Two of the three contaminated duodenoscopes still presented residual contamination after three post-reprocessing procedures; the level of residual contamination was, however, lower than that seen after the first post-reprocessing procedure, indicating that the procedure had gradually reduced, though not totally eliminated, the level of contamination.

**Figure 1:**
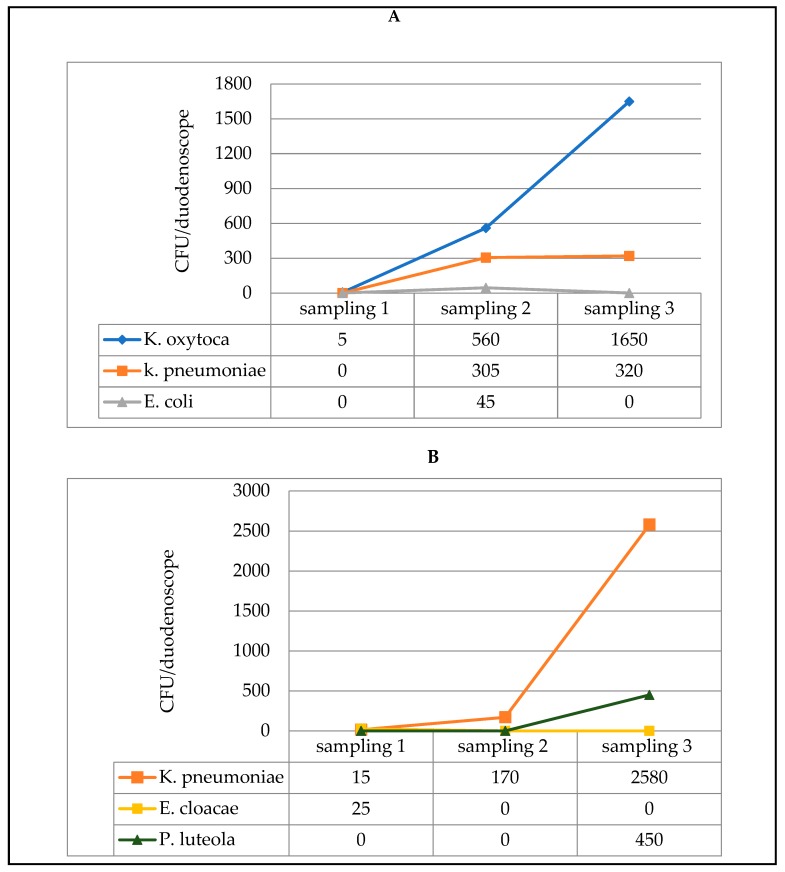
Microbial load measured in three samples, each of which was taken after a reprocessing procedure (3 consecutive procedures on the same day), from one of the three contaminated duodenoscopes: distal end sampling (**A**) and instrument channel sampling (**B**).

In the third duodenoscope, by contrast, an opposite trend was observed; instead of diminishing after the three consecutive reprocessing treatments, contamination by *K. oxytoca* and *K. pneumoniae* (distal end) and by *K. pneumoniae* and *P. luteola* (instrument channel) actually increased (Figure 1).

The three contaminated duodenoscopes were returned to the manufacturer, who identified some problems—worn sheaths and damage to the end-cap—and carried out both internal and external decontamination of the devices. When the duodenoscopes were returned to the Endoscopy Unit, we verified the efficacy of this decontamination before the devices re-entered service.

During the period of inspection by the manufacturer, three replacement duodenoscopes were used; these underwent microbiological analyses over a 3-month period and proved suitable for use. Microbiological analysis of water from the last rinse always yielded negative results.

The findings of microbiological non-conformity were immediately communicated to the hospital’s Chief Information Officer, to the Medical Director, to the Head of the Endoscopy Unit, and to the Nursing Coordinator. In addition, epidemiological surveillance was undertaken on patients who had undergone ERCP in the week prior to the post-reprocessing microbiological monitoring of every device. During the audits carried out in June 2017 with the Endoscopy Unit staff responsible for reprocessing, and during on-site inspections, some shortcomings emerged, especially in the pre-cleaning procedure, leak testing, and manual brushing. On-the-job staff training was, therefore, undertaken and staff members were urged to pay special attention to the phases of reprocessing that had proved particularly problematic and inadequate. Certain modifications were also recommended, including the replacement of some equipment, such as endoscope washers and device storage lockers, and restructuring of the manual washing area; these modifications were implemented in January 2019.

**Figure 2:**
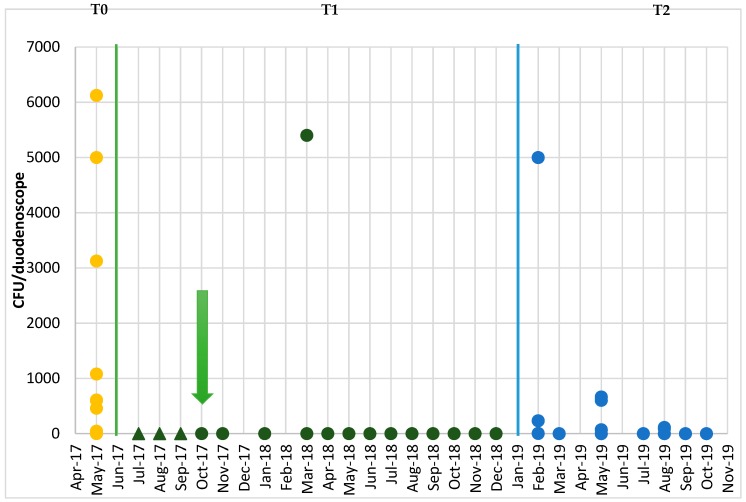
Temporal trend in the total concentrations (CFU/duodenoscope) recorded in the duodenoscopes throughout the period of microbiological surveillance. Green arrow: Date of return of the duodenoscopes from the manufacturing company. Dark green triangles: Substitute duodenoscopes. Dots: Duodenoscopes under surveillance.

Subsequently, a further series of sampling was undertaken in order to verify the efficacy of the corrective measures that had been implemented. The results of the post-reprocessing surveillance of the endoscopes at Time T1, after the audit with the personnel responsible for reprocessing (Figure 2), revealed only one case of non-conformity, in samples from both the distal end and the instrument channel; this concerned a duodenoscope, in which the presence of *K. oxytoca* was detected (2700 CFU/duodenoscope at the distal end and 2700 CFU/duodenoscope in the instrument channel). Subsequent reprocessing eliminated this contamination.

The further microbiological controls carried out in the months following Time T1 always yielded negative results.

Following modernisation of the equipment (purchase of new endoscope washers and new storage lockers, etc.) (Time T2), cases of non-conformity accounted for 29.41% of the samples taken from the distal end and for 47.06% of those taken in the channels. Specifically, in the positive samples from the distal end, *P. aeruginosa* was detected in a range from 12 to 2500 CFU/duodenoscope; with regard to the instrument channel, 35.29% of samples were contaminated by *P. aeruginosa* (range 4-2500 CFU/duodenoscope).

In this phase, the microbiological quality of the water was again assessed; as the results were negative, we were able to exclude the possibility that the water supply was the source of *P. aeruginosa*.

Figure 2 reports the temporal trend in the total concentrations (sum of the concentrations in samples from the distal end and instrument channel) (CFU/duodenoscope) recorded in the duodenoscopes throughout the period of microbiological surveillance (Times T0, T1 and T2).

## 4. Discussion

Despite advancements in knowledge and practice, endoscopic procedures may constitute a risk factor for the transmission of infections.

The risk of transmitting infections was formerly estimated to be 1 in 1.8 million endoscopic procedures. This figure now appears to be a significant underestimation for many reasons, including a lack of detailed surveillance for infections following endoscopy, under-reporting, and a lack of recognition of acknowledged transmissions [20,21].

A report on the top 10 health technology hazards of 2018 ranked failure to consistently and effectively reprocess flexible endoscopes as one of the biggest threats to health-care delivery and patient health, second only to threats to cybersecurity [2].

Recent years have seen the consolidation of scientific knowledge of the most appropriate modalities for treating endoscopes in such a way as to prevent the spread of infections (reprocessing of endoscopes). This knowledge has given rise to numerous guidelines, drawn up by individual institutions, scientific societies or, more recently, groups of scientific societies and representatives of the medical industry, who have issued joint recommendations based on systematic reviews of the literature [1].

Several studies have demonstrated that rigorous adherence to the various guidelines regarding the reprocessing of endoscopes is the principal means of preventing cross-transmission.

Others, however, have found that, despite the prescribed procedures, some manual phases of pre-treatment, decontamination, and cleansing, which are strictly operator-dependent, are frequently neglected or improperly carried out. Other reasons for persistent contamination may be acquired and inherent endoscope defects, inappropriate or defective cleaning supplies, and biofilm formation [21,22,23].

In addition, there are problems related to the particular structure of the endoscope; duodenoscopes, for example, are equipped with an elevator channel (Albarran), a single channel that enables the patient’s biliary tract to be visualised, and through which guide wires, biliary catheters, and stents are inserted. This channel has a very complex structure and is sometimes very difficult to disinfect completely by means of the traditional reprocessing techniques.

The recent introduction of automatic endoscope washers that disinfect these devices has improved both operator performance and patient safety. Nevertheless, if biofilm is present inside the channels, the efficacy of the disinfection process is not always guaranteed.

Microbiological surveillance by means of culture-based methods is an established and easy-to-use approach to assessing the effectiveness of reprocessing procedures [5]. In this paper, we report the results of a microbiological surveillance study conducted over a period of three years in the Digestive Endoscopy Unit of an Italian hospital. The initial phase of surveillance, which involved the contemporary evaluation of the four duodenoscopes, revealed that three of these duodenoscopes presented a high level of contamination, both in terms of the concentration of high-concern micro-organisms detected (*P. aeruginosa*, *K. pneumoniae*, *K. oxytoca*, *Enterobacter spp*, *S. maltophilia*, *A. baumannii*, *E. coli*, *C. freundii*), and in terms of the percentage of positive samples, with concentrations that, in some cases, reached values of 2600 CFU at the distal end and/or in the instrument channel.

Similar results were obtained in a study conducted by Ribeiro et al. [24], which evaluated contamination in reprocessed endoscopes (gastroscopes and colonoscopes). Contamination was detected in 71.8% (28/39) of the samples obtained from the air/water channels of colonoscopes, and in 70% (42/60) of the samples from the air/water channels of gastroscopes. The main micro-organisms that were isolated from the endoscopes were, *Pseudomonas aeruginosa*, *Escherichia coli*, *Acinetobacter baumannii*, and *Klebsiella pneumoniae*.

The growing phenomenon of antibiotic resistance in hospital environments [25,26,27,28,29,30,31,32], especially in Gram-negative bacteria, is well-known and is one of the most serious problems facing public health systems. In this regard, our results on the antibiotic resistance of the isolated strains are of particular interest, revealing the presence of multi-resistant strains in both distal end and instrument channel samples (*K. pneumoniae* ESBL+, *P. aeruginosa* resistant to multiple antibiotics).

The presence of multi-resistant strains in devices used for endoscopy may have serious consequences for health. In this regard, several serious outbreaks caused by multi-resistant micro-organisms following endoscopic procedures have been reported [33]. Aumeran et al. [34] reported a duodenoscope-associated outbreak with extended-spectrum beta lactamase (ESBL)-producing *K. pneumoniae*. Similarly, Bajolet et al. [35] reported an outbreak at a hospital in Reims, France, in 2011, which was traced to a single endoscope contaminated with ESBL-producing *P. aeruginosa*.

Recent studies have shown that iatrogenic effects during endoscopy include, not only mucosal damage, but also infections due to biofilm growth inside the endoscope, especially in immuno-compromised patients [36]. The fact that biofilms can form inside endoscopes was confirmed in the present study. Indeed, in one of the duodenoscopes monitored, microbial contamination was seen to increase, instead of gradually diminishing, after three reprocessing procedures; this was probably due to the detachment of portions of biofilm. Specifically, the concentration of *K. pneumoniae* in the instrument channel rose from 15 CFU to 170 CFU over the three post-reprocessing sampling sessions, reaching 2580 CFU at the end of the last reprocessing procedure.

As indicated by the CDC guidelines, the three contaminated duodenoscopes that we discovered were returned to the manufacturer for examination and decontamination. The findings of microbiological non-conformity were immediately communicated to the hospital’s Chief Information Officer, to the Medical Director, to the Head of the Endoscopy Unit, and to the Nursing Coordinator. In addition, epidemiological surveillance was undertaken on patients who had undergone ERCP in the week prior to the post-reprocessing microbiological monitoring of the duodenoscopes; this did not reveal any cases of infection that could be linked to the use of the endoscopes. However, as the epidemiological surveillance involved only hospitalised patients, and not outpatients, the result might have been underestimated. This constitutes a limitation of the study.

During the audits carried out with the Endoscopy Unit staff responsible for reprocessing, and during on-site inspections, some breaches emerged, especially in the manual cleaning of the endoscopes. Moreover, it was ascertained that some equipment, such as endoscope washers and device storage lockers, needed to be replaced, and that the manual washing area needed to be restructured; these measures were implemented in January 2019.

The results of the post-reprocessing surveillance of the endoscopes at Time T1, after the audits and on-the-job staff training, revealed only one case of non-conformity.

Finally, once the equipment had been modernised (Time T2), a sudden increase in cases of non-conformity was recorded (29.41% and 47.06% in samples taken from the distal end, and the instrument channel, respectively); the species chiefly involved was *P. aeruginosa*. Evaluation of the microbiological quality of the water carried out during the T2 phase yielded negative results, which enabled us to exclude the possibility that the water supply was the source of *P. aeruginosa*. A possible explanation of this increased contamination could be that staff compliance with the proper reprocessing procedures had declined, particularly with regard to the operator-dependent phases, such as manual cleansing. This reduced compliance probably stemmed from excessive reliance on the new equipment. Once these cases of non-conformity had been promptly signalled and staff had been reminded to take the utmost care in the various critical phases, no further non-conformity was recorded.

## 5. Conclusions

In conclusion, our study highlighted both the potential risk of transmitting pathogens through the use of duodenoscopes and the importance of implementing a well-structured system of microbiological surveillance and training programs, in order to improve the reprocessing protocol and reduce, as far as possible, the risk of spreading ERCP-associated infections.

In addition, the terminal cleaning process of the endoscopic unit that should include cleaning of surfaces in the procedure room is equally important because they constitute a possible transitory site for the accumulation of microorganisms [7,37].

## Figures and Tables

**Table 1:** Minimum and maximum concentrations of high-concern microorganisms (CFU/duodenoscope) and percentages of positivity of samples taken from the distal end and the instrument channel of the duodenoscopes.

	Distal End	Instrument Channel
Micro-Organism	Min-Max CFU/Duodenoscope (% Positivity)	Min-Max CFU/Duodenoscope (% Positivity)
*P. aeruginosa*	10–2500 (41.67)	15–2500 (41.67)
*K. pneumoniae*	120–650 (41.67)	15–2580 (50.00)
*K. oxytoca*	5–1650 (25.00)	-
*Enterobacter spp*	-	25–50 (16.67)
*S. maltophilia*	20–25 (16.67)	20–120 (16.67)
*A. baumannii*	2600 (16.67)	360–2600 (16.67)
*E. coli*	45 (8.33)	-
*C. freundii*	125 (8.33)	-
